# Crassolide Suppresses Dendritic Cell Maturation and Attenuates Experimental Antiphospholipid Syndrome

**DOI:** 10.3390/molecules26092492

**Published:** 2021-04-24

**Authors:** Chi-Chien Lin, Yu-Kang Chang, Shih-Chao Lin, Jui-Hsin Su, Ya-Hsuan Chao, Kuo-Tung Tang

**Affiliations:** 1 Ph.D. Program in Translational Medicine, National Chung Hsing University, Taichung 402, Taiwan; lincc@email.nchu.edu.tw; 2 Institute of Biomedical Science, iEGG and Animal Biotechnology Center, National Chung-Hsing University, Taichung 402, Taiwan; demonsandy@gmail.com; 3 Department of Medical Research, China Medical University Hospital, Taichung 404, Taiwan; 4 Department of Medical Research, Taichung Veterans General Hospital, Taichung 407, Taiwan; 5 Department of Pharmacology, College of Medicine, Kaohsiung Medical University, Kaohsiung 807, Taiwan; 6 Department of Medical Research, Tungs’ Taichung Metro Harbor Hospital, Taichung 433, Taiwan; yogurt8306@gmail.com; 7 Department of Nursing, Jen-Teh Junior College of Medicine and Management, Miaoli 356, Taiwan; 8 Bachelor’s Degree Program in Marine Biotechnology, College of Life Sciences, National Taiwan Ocean University, Keelung 202, Taiwan; sclin@mail.ntou.edu.tw; 9 National Museum of Marine Biology and Aquarium, Pingtung 944, Taiwan; x2219@nmmba.gov.tw; 10 Division of Allergy, Immunology and Rheumatology, Taichung Veterans General Hospital, Taichung 407, Taiwan; 11 Faculty of Medicine, National Yang-Ming University, Taipei 112, Taiwan

**Keywords:** antiphospholipid syndrome (APS), autoimmune, β2-glycoprotein I, crassolide, soft corals, dendritic cells, T cells

## Abstract

Antiphospholipid syndrome (APS) is an autoimmune disease characterized by the production of β2-glycoprotein I (β2GPI)-dependent autoantibodies, with vascular thrombosis or obstetrical complications. Around 20% of APS patients are refractory to current treatments. Crassolide, a cembranoid diterpene extracted from soft corals, is a potential therapeutic candidate. Here, to examine the anti-inflammatory properties of crassolide, we first determined its effects on bone marrow-derived and splenic dendritic cells (DC). Specifically, we applied lipopolysaccharide (LPS) or β2GPI stimulation and measured the expressions of CD80 and CD86, and secretions of cytokines. We also determined in the OT-II mice, if bone marrow-derived DC was able to stimulate antigen-specific T cells. Moreover, we examined the therapeutic potential of crassolide postimmunization in a murine model of APS that depended on active immunization with β2GPI. The vascular manifestations were evaluated in terms of fluorescein-induced thrombi in mesenteric microvessels, whereas the obstetric manifestations were evaluated based on the proportion of fetal loss after pregnancy. We also measured blood titers of anti-β2GPI antibody, splenic cell proliferative responses and cytokine secretions after β2GPI stimulation ex vivo. Finally, we determined in these mice, hematological, hepatic and renal toxicities of crassolide. Crassolide after LPS stimulation suppressed DC maturation and secretion of tumor necrosis factor (TNF)-α, interleukin (IL)-6, IL-12 and IL-23, and downstream T cell activation. Crassolide could partially ameliorate both the vascular and obstetric manifestations of APS in BALB/c mice. Both blood titers of anti-β2GPI antibody and splenic cell proliferation after β2GPI stimulation were reduced. Splenic Th1 and Th17 responses were also lowered after β2GPI stimulation. Finally, within therapeutic doses of crassolide, we found no evidence of its toxicity. In conclusion, we showed the ability of crassolide to suppress DC and downstream T cell responses. Crassolide is therefore a potential candidate for adjunctive therapy in APS.

## 1. Introduction

Antiphospholipid syndrome (APS) is an autoimmune disease characterized by the production of β2-glycoprotein I (β2GPI)-dependent antiphopholipid autoantibodies (like APL, including lupus anticoagulant, anticardiolipin antibody, anti-β2GPI antibody), arterial/venous thrombosis or obstetrical complications [1]. Its pathogenesis involves autoreactive T cells and B cells that produce autoantibodies against proteins like β2GPI [2]. Vascular APS is associated with severe morbidities like stroke, ischemic bowel disease, and even mortality, creating a significant disease burden for these patients [3]. Obstetric APS contributes to morbidity in both mother and fetus [4]. Treatment of APS is far from satisfactory [5]. Current treatment paradigm relies on antiplatelet agents and anticoagulants [6]. About 20% of patients with vascular APS develop recurrent thrombosis despite such standard therapy [7]. Current therapy also fails in 20–30% of patients with obstetric APS [8]. In addition, such treatment strategy inevitably carries the risk of hemorrhage [9], with annual major bleeding rates between 0.57 and 10% [10]. These shortcomings indicate the need for developing new adjunctive therapeutic interventions for this devastating disease.

Marine microorganisms contain resourceful bioactive molecules to be identified. Currently, a few promising marine compounds have been discovered and undergone the extensive clinical investigation for therapeutic uses against various diseases. For example, ziconotide (Prialt^®^, TerSera Therapeutics, Lake Forest, IL, USA), originated from a marine snail, *Conus magus*, was approved by US Food and Drug Administration (USFDA) in 2004 to manage pain in patients due to its capability to block the N-type calcium channel on spinal cords [11]. In our previous research, we have identified and characterized two distinct compounds from soft corals *Sinularia flexibilis* and *Lobophytum crissum* to potentially treat small-cell lung cancer and inflammation, respectively [12,13]. To address the lack of potent therapy for APS, we therefore turned to the marine organisms for seeking an alternative remedy.

**Figure 1:**
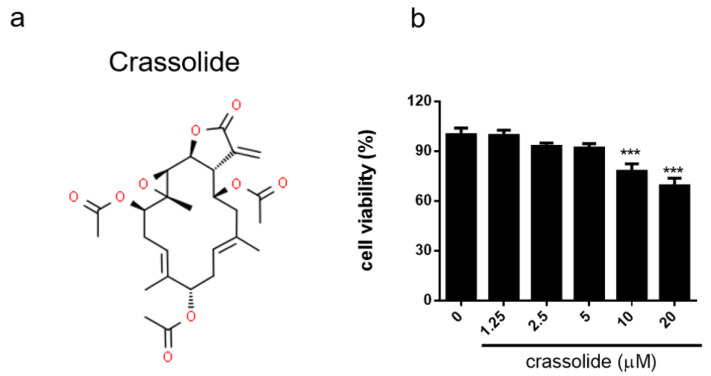
Effects of crassolide on BMDCs cell viability: (**a**) Chemical structure of crassolide. (**b**) Cell viability measured by CCK-8 viability assay after 24 h of crassolide treatment. Data are presented as percentages of the control treated with 0.1% DMSO. Values represent the means ± SEM from one of three independent experiments. *** *p* < 0.001, versus 0.1% DMSO-treated control group, tested by one-way ANOVA followed by Tukey’s multiple comparison test.

Crassolide, with a unique structural scaffold (Figure 1a), is one of the most abundant cembranoid diterpenes extracted from corals like *Sarcophyton crassocaule* and *Lobophytum crassum* and its bioactivities have been well-characterized [14,15]. Cembranoid diterpenes such as crassolide have been shown to possess potent anti-inflammatory activity through regulating, among other mechanisms, inducible nitric oxide synthase (iNOS), cyclooxygenase-2 (COX-2), or reactive oxygen species [12,16,17] as well as immunomodulatory activity through changing the status of bone marrow-derived dendritic cells (BMDCs) [13]. Also, DCs have been implied to be linked with the immunopathogenesis of APS and exacerbation of the severity of APS. Generally, it is proposed that oxidized β2GPI and/or anti-β2GPI antibodies bind to and activate DCs via toll-like receptor 4 (TLR-4), which could occur synergistically with the activation of DCs by apoptotic cells via TLR-7 or -8 [18,19]. With this concept in mind, it is not surprising to learn that tolerogenic DCs to β2GPI antigen can lower the severity of APS [20]. Due to the pivotal role that DCs play in APS, we aimed to design a series of experiments to investigate therapeutic effect of crassolide on APS and determine whether crassolide can harmonize DC functions in an APS mouse model.

## 2. Results

### 2.1. No Discernible Toxicity of Crassolide

To evaluate the effect of crassolide (Figure 1a) on DCs, we first determined its cytotoxicity on mouse BMDCs using the CCK-8 assay. Figure 1b shows the cell viability results on BMDCs. Viability remained >90% after 5 μM crassolide treatment. The similar effects of crassolide were determined at doses from 1.25 to 5 μM crassolide. We had conducted a dose toxicity test in mice. Results showed that crassolide, when applied only at doses of 10 and 20 mg/kg, had produced hematotoxicity, hepatotoxicity and renotoxicity, but not at 5 mg/kg (Supplementary Figure S1). Therefore, we chose crassolide 5 mg/kg for the following in vivo experiments.

### 2.2. Crassolide Lowered LPS-Induced Expressions of Co-Stimulatory Molecules and Secretions of Pro-Inflammatory Cytokines in BMDCs

To investigate the crassolide influences on phenotypic maturation of DC, BMDCs were stimulated with LPS, a TLR4 agonist, together with or without crassolide. Expressions of co-stimulatory molecules, CD40, CD80, and CD86, were then determined with flow cytometry.

**Figure 2:**
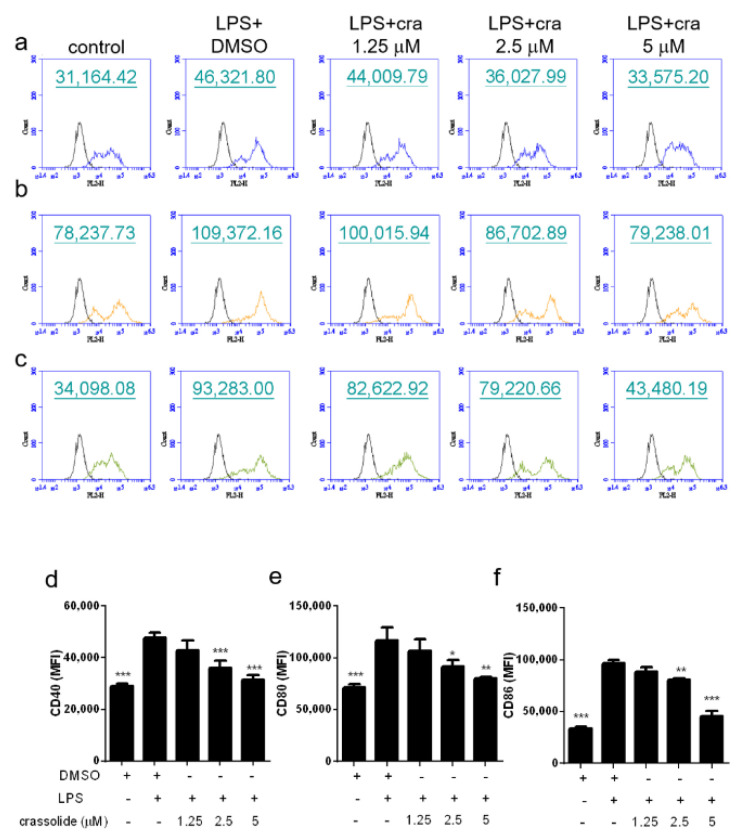
Effects of crassolide on lipopolysaccharides (LPS)-induced expression of surface markers in bone marrow-derived dendritic cells (BMDCs). BMDCs were treated for 2 h with either different doses of crassolide, or 0.1% DMSO followed by LPS treatment (100 ng/mL) for 24 h. (**a**–**c**) Expression levels of CD40 (**a**), CD80 (**b**) and CD86 (**c**) measured by flow cytometry. CD11c was used to gate live DCs. The black line represents cells labeled with isotype-matched control antibodies. (**d**–**f**) The relative mean fluorescence intensity (MFI) ± SEM of triplicate samples from one of three independent experiments. * *p* < 0.05, ** *p* < 0.01, *** *p* < 0.001, versus LPS + 0.1% DMSO-treated group, tested by one-way ANOVA followed by Tukey’s multiple comparison test.

**Figure 3:**
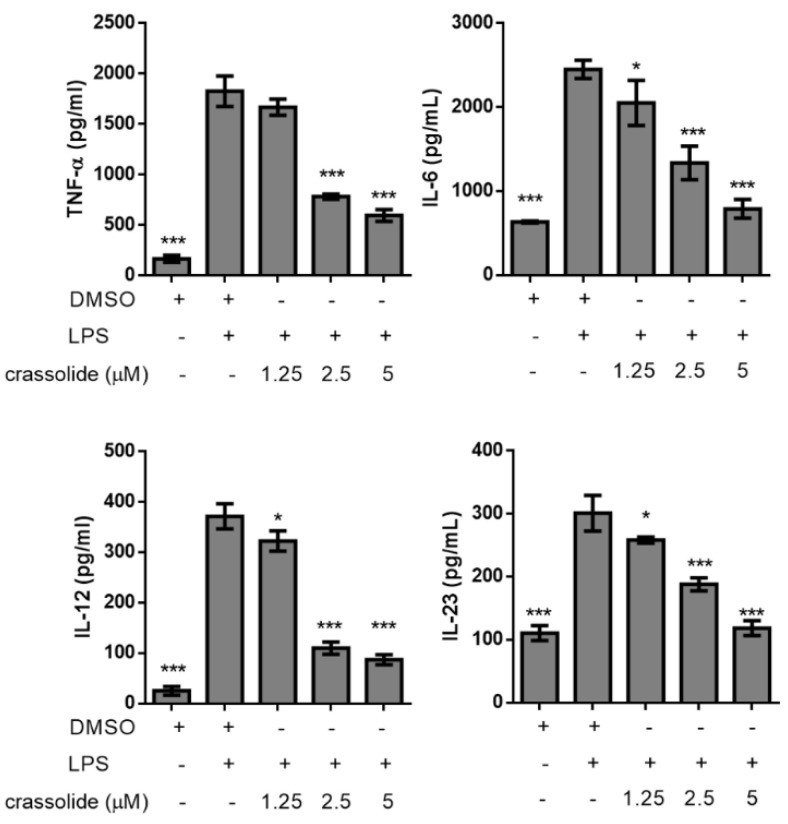
Effects of crassolide on lipopolysaccharides (LPS)-induced cytokines production in bone marrow-derived dendritic cells (BMDCs). BMDCs were treated for 2 h with either various doses of crassolide, or 0.1% DMSO, followed by LPS stimulation (100 ng/mL) for 24 h. Cytokines in the culture supernatants were measured by ELISA. Data are presented as mean ± SEM of triplicate samples from one of three independent experiments. * *p* < 0.05, *** *p* < 0.001, versus LPS + 0.1% DMSO-treated group, tested by one-way ANOVA followed by Tukey’s multiple comparison test.

As shown in Figure 2, LPS increased expressions of CD40 (Figure 2a,d), CD80 (Figure 2b,e), and CD86 (Figure 2c,f) of BMDCs. Such effects were significantly attenuated by crassolide in a dose-dependent manner. Furthermore, we determined secretions of cytokines in BMDCs after LPS stimulation. Results suggested that LPS stimulation had enhanced the production of pro-inflammatory cytokines like TNF-α, IL-6, IL-12 and IL-23. Such increased cytokine productions were blocked by crassolide in a dose-dependent way (Figure 3). Taken together, findings indicated that crassolide suppressed DC maturation, with effects manifested as a weaker expression of co-stimulatory molecules and production of pro-inflammatory cytokines.

### 2.3. Crassolide Suppressed LPS-Induced Antigen-Specific T Cell Proliferation and Cytokines Production in DCs

**Figure 4:**
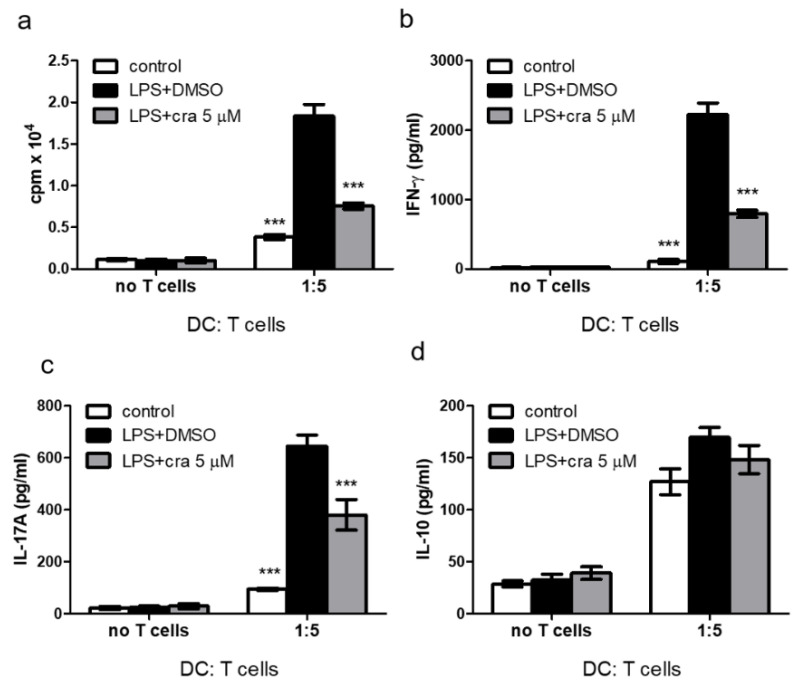
Effects of crassolide on the ability of lipopolysaccharides (LPS)-stimulated BMDCs from OT-II mice, which produce CD4+ T cells which recognizes OVA_323–339_, to induce antigen-specific CD4+ T cell proliferation and cytokines production. (**a**) Cell proliferation of OT-II CD4+ T cells measured by ^3^H-TdR incorporation assay after co-culturing with OVA_323–339_ (OVAP_2_) peptide and LPS-stimulated BMDCs in a 5:1 ratio with or without crassolide treatment for 96 h. (**b**–**d**) The amount of cytokines in the culture supernatants measured by ELISA. Data are presented as mean ± SEM of triplicate samples from one of three independent experiments. *** *p* < 0.001, versus LPS + 0.1% DMSO-treated group, tested by one-way ANOVA followed by Tukey’s multiple comparison test.

OT-II mice produce major histocompatibility complex (MHC) class II-restricted CD4+ T cells which recognizes OVA_323– 339_ and expresses the αβ T cell receptor (TCR), Vα2, and Vβ5 [21,22]. We further studied effects of crassolide on DC-mediated antigen-specific T cell responses in vitro. BMDCs from OT-II mice were pre-loaded with the OVA_323–339_ (OVAP_2_) peptide and stimulated for 24 h with LPS in the presence or absence of crassolide (5 μM). The different groups of DCs were co-cultured for 96 h with splenic CD4+ T cells extracted from OT-II mice at a cell ratio of 1:5. Figure 4a shows LPS-treated DCs showed an enhanced ability to stimulate OT-II-specific CD4+ T cells proliferation in the absence of crassolide. Co-culturing with crassolide-treated DCs, on the other hand, suppressed the proliferation of OT-II-specific CD4+ T cells. Concomitantly, crassolide treatment also reduced levels of IFN-γ (Figure 4b) and IL-17A (Figure 4c), as detected in the culture supernatants. Findings were consistent with the activation of Th1 and Th17 lineage. IL-10 production was however unaffected by crassolide treatment (Figure 4d). Thus, in vitro, crassolide had suppressed DC-stimulated antigen-specific T cell proliferation and cytokines production.

**Figure 5:**
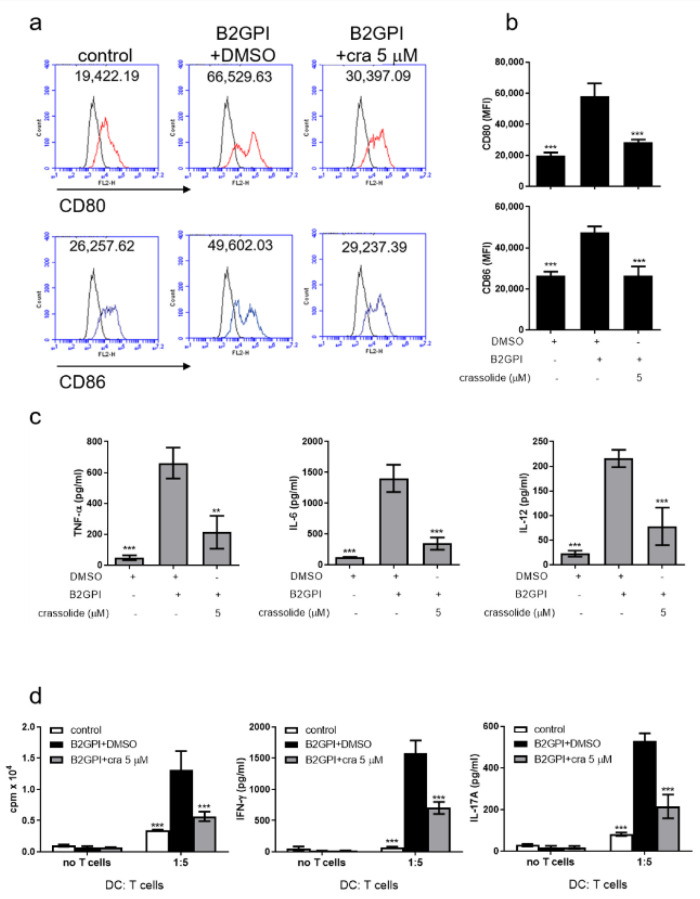
Effects of crassolide on the β2GPI-stimulated BMDCs and T cells. (**a**) and (**b**) CD80 and CD86 expressions of BMDCs from bone marrows of naïve BALB/c mice were analyzed with flow cytometry. (**c**) TNF-α, IL-6, and IL-12 from BMDC cultured supernatant were measured by ELISA with or without crassolide treatment. (**d**) Cell proliferation and T-cell lineage cytokines of OT-II CD4+ T cells from OT-II mice was measured by ^3^H-TdR incorporation assay after co-culturing with OVA_323–339_ (OVAP_2_) peptide and β2GPI-stimulated BMDCs with or without crassolide treatment for 96 h. Data are presented as mean ± SEM of triplicate samples from one of three independent experiments. ** *p* < 0.01; *** *p* < 0.001, versus β2GPI + 0.1% DMSO vehicle group and calculated with one-way ANOVA followed by Tukey’s multiple comparison test.

Given β2GPI is the major antigen for autoantibodies that highly involve in the APS, we therefore treated BMDCs and T cells with 50 μg/mL of β2GPI under the same conditions as previous LPS treatments and measured the co-stimulatory molecule expressions as well as the cytokine productions from BMDCs and T cells. As the results shown in Figure 5, the increased expressions of CD80 and CD86 and elevated cytokines, including Th1 and Th17-related cytokines, were significantly reduced by crassolide. The data here are consistent with our previous findings observed in the LPS model, confirming the anti-APS effect of crassolide. 

### 2.4. Crassolide Inhibited IRAK-1 Phosphorylation and NF-κB Activation in BMDCs

**Figure 6:**
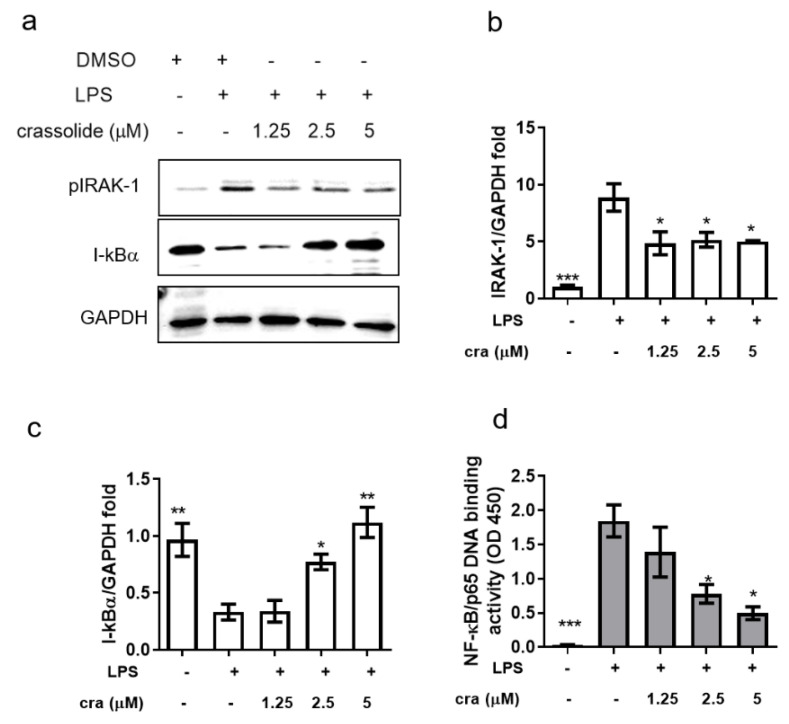
Effects of crassolide on the pIRAK-1 and IkBα expression and p65 nuclear translocation in BMDC stimulated with LPS. BMDCs were treated for 2 h with either various doses of crassolide or 0.1% DMSO, followed by LPS stimulation (100 ng/mL) for 6 h. (**a**) Cells were harvested and total protein was extracted for Western blotting assay to detect pIRAK-1, and IkB protein levels. (**b**,**c**) GAPDH was used as a loading control, and the quantified expression levels by ImageJ software were plotted in the bar graphs. (**d**) NF-κB p65 DNA-binding activity in nuclear extracts of BMDC cells was determined using the TransAM kit, with the optical density at 450 nm (OD450). Data are presented as mean ± SEM of triplicate samples from one of three independent experiments. * *p* < 0.01, ** *p* < 0.01, *** *p* < 0.001, versus LPS + 0.1% DMSO-treated group, tested by one way ANOVA followed by Tukey’s multiple comparison test.

The importance of interleukin-1 receptor associated kinase (IRAK)/nuclear factor kappa-light-chain-enhancer of activated B (NF-κB) signaling in human DCs after β2GPI stimulation has been demonstrated in a prior study [18]. In order to investigate whether crassolide-mediated inhibition of inflammatory response in BMDCs resulted from NF-κB signaling inactivation, Western blotting and DNA binding assay were performed to measure IRAK-1 phosphorylation, IkBα (corepressors of NF-κB) expression and p65 nuclear translocation. LPS stimulation significantly induced IRAK-1 phosphorylation (Figure 6a,b) and decreased the IkBα (Figure 6a,c) expression, which could be restored in the presence of crassolide (Figure 6a–c). p65 resided in the cytoplasm in the control group, and a translocation to the nucleus was observed after stimulation with LPS. Crassolide treatment retained p65 in the cytoplasm (Figure 6d).

### 2.5. Therapeutic Effects of Crassolide on APS Manifestations

**Figure 7:**
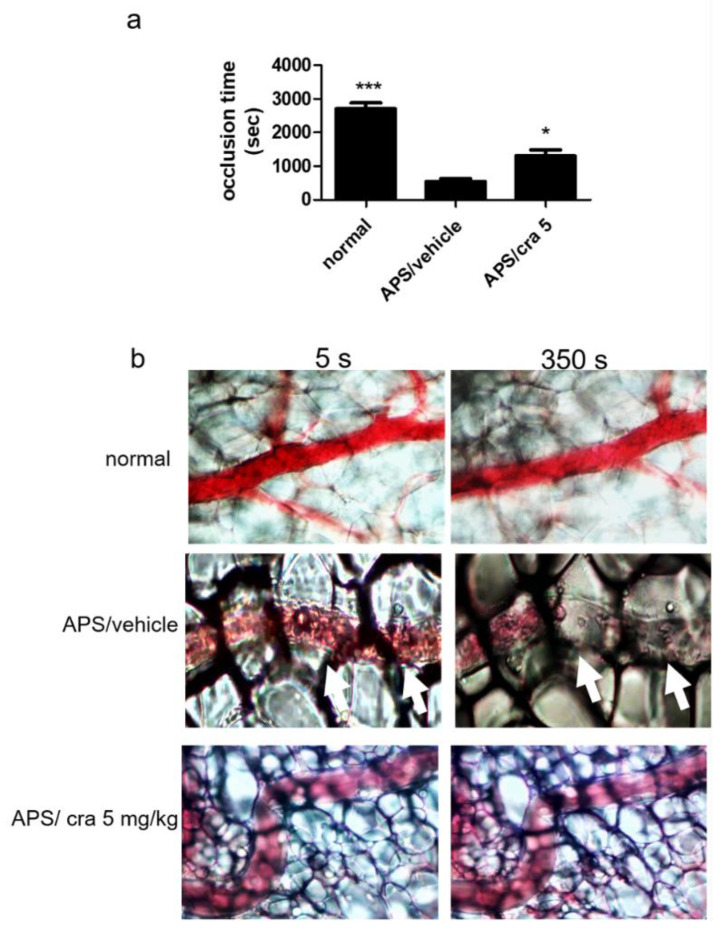
Effects of crassolide on thrombosis formation in the mesenteric venules of mice. Mice were given vehicle control (10% DMSO and 90% glyceryl trioctanoate) or crassolide (5 mg/kg) and the mesenteric venules were irradiated to induce microthrombus. (**a**) The occlusion time, (**b**) Microscopic images (400× magnification). Data are presented as mean ± SEM of 5 mice from one of three independent experiments. * *p* < 0.05, *** *p* < 0.001, versus vehicle-treated APS mice group, tested by one-way ANOVA followed by Tukey’s multiple comparison test.

To examine the immunomodulatory effects of crassolide in vivo, the β2GPI-immunized mouse model of APS was used to assess the therapeutic effects of crassolide on APS manifestations. Table 1 shows the findings: 5 mg/kg crassolide produced less severe APS manifestations as follows: prolonged aPTT, decreased platelet count, anti-β2GPI antibody, increased percentage of fetal loss, and decreased mesenteric venule occlusion time. In particular, the mesenteric venule occlusion time was prolonged by ~2 fold (858 ± 493 s) when compared with controls (538 ± 202 s) (Figure 7a,b).

### 2.6. Crassolide Suppressed Splenic DC Maturation and Cytokines Production in APS Mice

**Figure 8:**
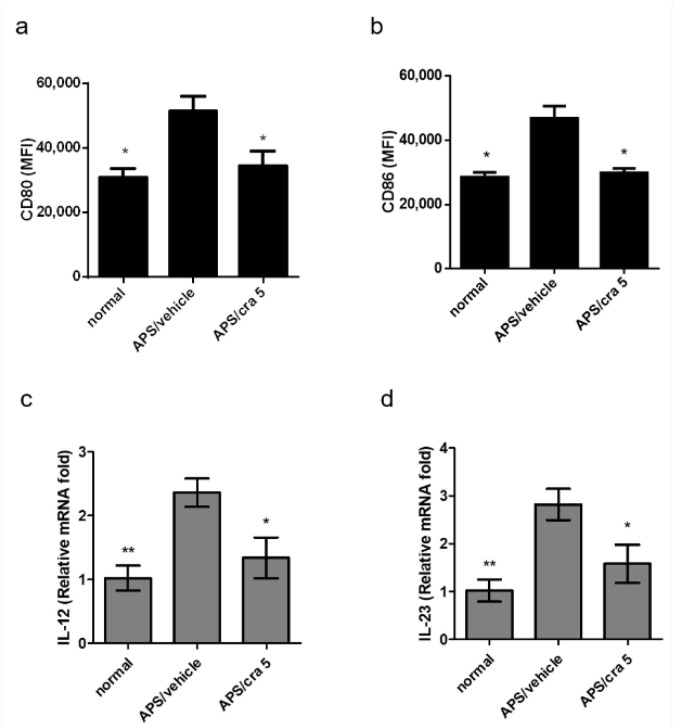
Effects of carssolide on the expression of surface markers of splenic DCs in APS mice. Spleen cells were purified from different mouse groups on day 56, and the relative mean fluorescence intensity (MFI) of (**a**) CD80 and (**b**) CD86 were measured by flow cytometry. Data are presented as mean ± SEM of 5 mice from one of three independent experiments. (**c**) IL-12 and (**d**) IL-23 mRNA expression levels in purified splenic CD11c+DCs determined by reverse transcription-quantitative PCR. Data were normalized to hypoxanthine guanine phosphoribosyl transferase 1 expression levels. * *p* < 0.05, ** *p* < 0.01, versus vehicle-treated APS mice group, tested by one way ANOVA followed by Tukey’s multiple comparison test.

We further determined whether crassolide modulates the maturation of splenic DCs in APS mice. In comparison with vehicle-treated APS controls, 5 mg/kg crassolide treatment significantly reduced the expression of CD80 (Figure 8a) and CD86 (Figure 8b) in CD11c+DCs (Supplemental Figure S2 and Figure 8a,b). In addition, we further found, using RT-PCR, lower expression levels of IL-12 (Figure 8c) and IL-23 (Figure 8d) in the crassolide-treated animals.

### 2.7. Crassolide Suppressed Β2GPI-Specific Splenic Th1 and Th17 Responses in APS Mice

**Figure 9:**
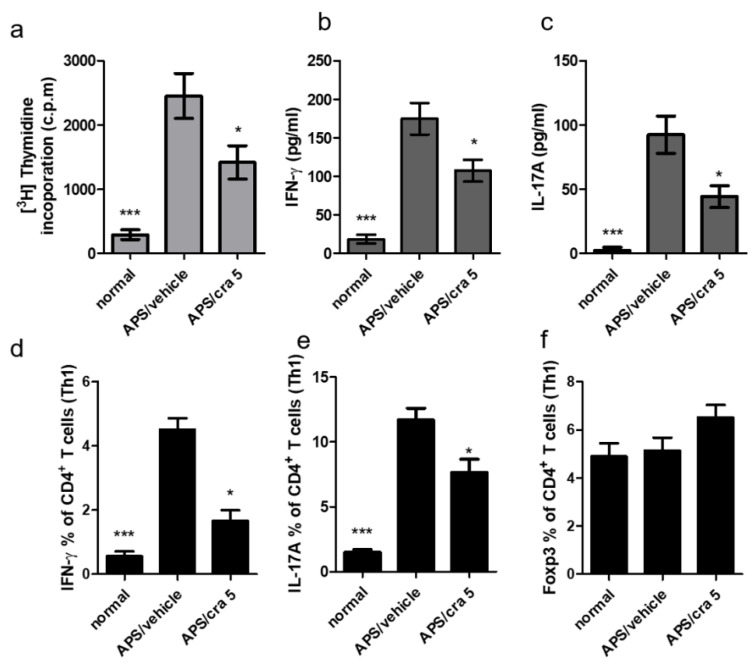
Effects of crassolide on cell proliferation and cytokines production in the spleen CD4+ T cells of APS mice after Β2GPI stimulation. (**a**) Spleen was harvested on day 56 and cultured in the presence of Β2GPI for 96 h. (**a**) Proliferation determined by radioactivity incorporation in count per minute (cpm) after pulsed treatment with 1 μCi of [^3^H]thymidine per well for the last 18 h. (**b**,**c**) The amounts of (**b**) IFN-γ and (**c**) IL-17A in the culture supernatants measured by ELISA. (**d**,**e**) The percentages of (**d**) IFN-γ+, (**e**) IL-17A+ and (**f**) Foxp3+ cells among CD4+ T cells measured by flow cytometry. Data are presented as mean ± SEM of 5 mice from one of three independent experiments. * *p* < 0.05, *** *p* < 0.001, versus vehicle-treated APS mice group, tested by one-way ANOVA followed by Tukey’s multiple comparison test.

Spleen cells were first isolated from different experimental groups and incubated with β2GPI for 96 h. Figure 9a shows the reduced proliferative response of splenic cells after β2GPI stimulation in crassolide-treated APS mice compared with vehicle-treated controls. In addition, levels of IFN-γ (Figure 9b) and IL-17A (Figure 9c) as detected in the supernatants of cultured splenocytes from crassolide-treated mice were significantly lower than those from the vehicle-treated controls. Flow cytometry also revealed significantly fewer CD4+ IFN-γ+ Th1 (Figure 9d) and CD4+ IL-17 + Th17 (Figure 9e) cells in the crassolide-treated group when compared with vehicle-treated controls (Supplemental Figure S3 and Figure 9d,e). In addition, we examined the percentage of Fopx3+CD4+ Treg cells but found no difference between the crassolide-treated group and vehicle-treated controls (Figure 9f and Supplementary Figure S3).

## 3. Discussion

We have conducted the first study on the therapeutic potential of crassolide, a cembranoid diterpene extracted from soft corals, in a murine model of APS. Results showed that crassolide ameliorated both the vascular and obstetric manifestations of APS in mice. This beneficial effect was mediated partly through the suppressed response of DC after stimulation, and its downstream T cell activation. Furthermore, the effective dose of crassolide produced no hematological, hepatic or renal toxicity in mice. Crassolide may be a potential therapeutic candidate for APS.

Previous studies had demonstrated that cembranoid diterpenes exerted a myriad of anti-inflammatory effects, which included suppressed NO production and cyclooxygenase (COX)-2 expression in mouse macrophages [16,23,24,25,26]. Effects also included the inhibitions of superoxide generation and elastase release in human neutrophils, after *N*-formyl-methionyl-leucyl-phenyl-alanine (fMLP) stimulation [27,28,29], and the attenuation of IL-12 and NO productions in DC after LPS stimulation [12]. Our earlier work on DC evaluated the effect of sinulariolide, another cembranoid diterpene derived from *Sinularia flexibilis* [30]. We found that sinulariolide had suppressed DC activation and the release of tumor necrosis factor (TNF)-α, IL-6, IL-12 and NO after LPS stimulation, partly through repressing signaling via NF-κB. In addition, the ability of DC to stimulate allogeneic CD4+ T cell proliferation was reduced by sinulariolide [31]. Radhika et al., examined the in vivo anti-inflammatory effect of these compounds [17]. They found that both acute inflammation in carrageenan-induced rat hind paw edema and chronic inflammation in cotton pellet-induced granuloma could be reduced by lobohedleolide, also a cembranoid diterpene. However, these compounds have not yet been used to treat any experimental animal model of autoimmune diseases. Our results supported the therapeutic potential of crassolide, a cembranoid diterpene, in both vascular and obstetric manifestations of APS in mice. While crassolide treatment did not fully reverse APS manifestations, its potential as an adjunctive therapy in APS should be further investigated. Another concern was its cytotoxicity, which is frequently observed with respect to cembranoid diterpenes [32]. Here, we did not find toxicity against crassolide-treated DCs. We also examined hematological, hepatic and renal toxicity of crassolide in mice. We found no evidence of any such concern at the therapeutic dose we used. Though promising, our preliminary results require further confirmation in both animals and humans. In addition, we need to conduct experiments to investigate the effects of crassolide upon endothelial cells activated by β2GPI due to the essential role of these cells in the pathogenesis of APS.

DC is pivotal in the pathogenesis of APS. Not only DC participates in its generation [33] but also in its propagation. Oxidized β2GPI, which shares the conformational change with autoantibody-bound β2GPI, activates human DC and the downstream Th1 response [18]. When autologous DCs from APS patients had been pulsed-treated with phospholipid-bound β2GPI, autoreactive T-cells were activated in vitro [34]. Moreover, tolerogenic DC, specific for β2GPI, had been induced by β2GPI and attenuated APS in mice [20], and in humans, CD4+ T cell response was also suppressed [35]. In the present study, we found that crassolide inhibited DC activation after stimulation, including expression of co-stimulatory molecules and secretion of inflammatory cytokines. This effect was observed in both LPS-treated BMDC from normal mice, and splenic DC from APS mice. In addition, we demonstrated that crassolide suppressed the ability of LPS-stimulated DC to activate antigen-specific T cells. Although we did not conduct experiments in β2GPI-stimulated BMDCs, our results implied that crassolide could suppress DC function and downstream T cell activation in APS in vivo. The speculation was based on our experimental findings in APS mice: (1). Crassolide treatment suppressed activation of splenic DC (expression of CD80 and CD86, and production of IL12 and IL23); (2). Crassolide treatment suppressed splenic Th1 and Th17 responses after β2GPI stimulation of extracted splenic cells. Moreover, our results in BMDCs after LPS stimulation implied that crassolide could suppress DC activation through the inhibition of IRAK/NF-kB signaling. These findings were consistent with the prior study, in which the crucial role of IRAK/NF-kB signaling in human DCs after β2GPI stimulation was demonstrated [18]. To be noted, we found that crassolide appeared to be more potent at DC modulation than sinulariolide, another diterpene, with the effective concentrations being 2.5 μM and 18.6 μM respectively [31]. In summary, crassolide suppressed DC activation both phenotypically and functionally and this effect may contribute to the amelioration of APS manifestations in mice.

T cells also contribute to the generation of APS. Previous studies reported increased Th1 and Th17 responses in APS patients [36,37,38]. Our earlier work also found increased Th1 and Th17 responses in APS mice. Such responses were suppressed along with ameliorated APS manifestations after DNA vaccination together with FK506 treatment [39]. In the present study, we noted a similar suppression of Th1 and Th17 responses in splenic T cells, as well as ameliorated APS manifestations in mice. Taken together, Th1 and Th17 responses likely participate in the pathogenesis of APS and their abolition may, at least partially, reverse it.

## 4. Material and Methods

### 4.1. Animals

Our study was approved by the Institutional Animal Care and Use Committee (IACUC) at National Chung Hsing University (serial number IACUC 106118). In line with our regulations, experimental mice were raised under specific pathogen-free (SPF)-free facilities, and were allowed free access to food and water. Breeding environment was controlled at 22 ± 2 °C, 60 ± 10% humidity, and under a 12/12 h light/dark illumination cycle. BALB/c female mice (6-weeks old) were provided by the National Laboratory Animal Center of Taiwan. OT-II TCR transgenic mice were provided by Dr. Ching-Liang Chu of the National Taiwan University, Taiwan.

### 4.2. Preparation of Bone Marrow-Derived Dendritic Cells (BMDCs)

BALB/c or OT-II mice were sacrificed with their femurs rapidly harvested and washed in sterile phosphate-buffered saline (PBS) to collect bone marrow cells. Marrow cells were extracted with an RBC lysis buffer to remove red blood cells. Cells were subsequently cultured in the RPMI 1640 growth medium for 7 days in a humidified 5% CO_2_ incubator at 37 °C. The growth medium contained also the following: l-glutamine, sodium pyruvate, 2-mercaptoethanol, 1% penicillin/streptomycin, 10% fetal bovine serum (FBS), and 10 ng/mL recombinant granulocyte-macrophage colony-stimulating factor (GM-CSF). Additional medium supplements (2 mL with 10 ng/mL GM-CSF) were supplied on days 3 and 5. By day 7, the nonadherent and loosely adherent cells were collected and taken as immature BMDCs, which were confirmed by an Accuri 5 flow cytometer (BD Biosciences, San Jose, CA, USA) based on their expression of CD11c. Afterwards, immature BMDCs were separated by CD11c microbeads (Miltenyi Biotec, Auburn, CA, USA) to obtain cell populations with >90% of CD11c+ BMDCs.

### 4.3. Chemicals

Crassolide was obtained from Dr Jui-Hsin Su (National Museum of Marine Biology and Aquarium, Pingtung, Taiwan), and was isolated from wild-type soft coral *Lobophytum crassum*. The stock solution was prepared at a concentration of 20 mg/mL in dimethyl sulfoxide (DMSO) (Sigma-Aldrich, St. Louis, MO, USA). The working solution was prepared by dilution with medium to desired concentrations. The purity of crassolide was 100% based on 1H-NMR and mass spectral analyses.

### 4.4. Cell Viability Assay

Murine BMDCs (2 × 10^5^ cells/mL) were cultured with crassolide at different concentrations. The vehicle control group was treated with 0.1% DMSO solvent for 24 h. Cell viability was determined using Cell Counting Kit-8 reagent (CCK-8, Sigma-Aldrich) per the manufacturer’s protocol.

### 4.5. Flow Cytometric Analyses of Surface Makers on Both BMDCs In Vitro and Splenic DCs Ex Vivo

Murine BMDCs (1 × 10^6^ cells/mL) from BALB/c, were pre-treated for 2 h with crassolide at various concentrations (1.25–5 μM) and then stimulated for 24 h with 100 ng/mL lipopolysaccharides (LPS) or 50 μg/mL β2GPI (After stimulation, cells were re-suspended in 500 μL of staining buffer (2% FCS and 0.05% NaN3 in PBS), and then stained on the ice for 45 min with mouse specific antibodies of CD11c (FITC-labeled), CD40, CD80 and CD86 (PE-labeled). After fixation, fluorescent intensities of CD40, CD80, and CD86 were determined using the Accuri 5 flow cytometer following gating with forward side scatter (FSC) and CD11c+ expression. Spleens of BALB/c mice were collected and then treated with RBC lysis buffer (Sigma-Aldrich) and passed through stainless 50 mesh to obtain single-cell suspensions. The aforementioned primary antibodies targeting cellular markers of DC were diluted in 500 μL of staining buffer for reaction at 4 °C overnight. These markers were analyzed using the Accuri 5 flow cytometer under the gating of FSC and CD11c. Data were expressed as mean fluorescence intensity (MFI).

### 4.6. Analyses of Cytokine Expressions

The levels of tumor necrosis factor (TNF)-α, interleukin (IL)-6, IL-12, and IL-23 present in cell culture supernatants were measured by sandwich ELISA (eBioscience, San Diego, CA, USA). Murine BDMCs (1 × 10^6^ cells/mL) from BALB/c were pre-treated for 2 h with crassolide at different concentrations (1.25–5 μM), and then stimulated for 24 h with 100 ng/mL LPS (Sigma-Aldrich) or 50 μg/mL β2GPI (Invitrogen, Rockford, IL, USA). The vehicle control cells were treated with 0.1% DMSO. Concentrations of cytokines were estimated based on standard curves according to manufacturer’s recommendations.

### 4.7. Analyses of OT-II T Cell Activation

Synthesized OVA_323–339_ (OT-II) peptides (10 μg/mL) were obtained from Echo Chemical Co. in Taiwan, and were added to CD11c+ BMDCs of OT-II mice and allowed reaction for 6 h, followed by the addition of 100 ng/mL LPS only or 50 μg/mL β2GPI, LPS or β2GPI plus 0.1% DMSO, or LPS plus crassolide for another 24 h. Ovalbumin-specific CD4+ T cells were purified from the splenocytes of OT-II mice using EasySep Mouse CD4+ T Cell Isolation Kit (Stem Cell, Grenoble, France). CD4+ T cells were co-cultured for 4 days with BMDCs at a ratio of 1:5 (DC: 5 × 10^4^ cells/well; T cell: 2.5 × 10^5^ cells/well). ^3^H-thymidine was added at 1 μCi/well for 18 h before cell harvest. Then, T cell proliferation was quantified based on the amount of incorporated radiolabeled ^3^H-thymidine, which was determined by the radioactivity in liquid scintillation counter (Beckman Instruments, Palo Alto, CA, USA). Supernatants from the co-culture of BMDC and OT-II T cells after a 96 h incubation period were further collected to measure levels of interferon (IFN)-γ, IL-17A, and IL-10 by ELISA (eBioscience) according to manufacturer’s recommendations.

### 4.8. Western Blotting

Murine BMDCs (1 × 10^6^ cells/mL) from BALB/c mice, were pre-treated for 2 h with crassolide at various concentrations (1.25–5 μM) and then stimulated for 12 h with 100 ng/mL lipopolysaccharides (LPS). The cells were harvested and cell pellets washed with ice-cold PBS. Centrifugated cell pellet were lysed on ice in RIPA lysis buffer (Cat# RP05-100, Visual Protein, Taipei City, Taiwan) containing 1% protease inhibitor cocktail (Sigma-Aldrich). Equal amounts of the protein were measured by the bicinchoninic acid (BCA) assay kit (Cat# BC03-500, Visual Protein). Normalized amounts of protein (30 μg) were loaded into each lane, separated by 10% SDS-PAGE at 100 V for 1.5 h, and transferred onto polyvinylidene difluoride (PVDF) membranes (Millipore, Billerica, MA, USA) by condition 300 mA for 1 h. We blocked the membranes with BlockPRO™ Protein-Free Blocking Buffer (Cat# BP01-1L, Visual Protein) for 1.5 h at room temperature, followed by incubation with the primary antibodies overnight at 4 °C. Western blot analyses with antibodies against anti-IRAK-1 (1:1000, clone D51G7, Cat# 4504S, Cell Signaling Technology, Danvers, MA, USA), anti-IkBα (1:1000, clone E130, Epitomics, Burlingame, CA, USA) and glyceraldehyde 3-phosphate dehydrogenase (GAPDH) (1:5000, clone 6C5, Cat# ab8245, Abcam, Cambridge, MA, USA) were performed. Subsequently, the membranes were incubated with horseradish peroxidase (HRP)-conjugated secondary antibody, and the immunoreactive bands developed with ECL reagent (GE Healthcare Life Sciences), which was visualized with the Hansor Luminescence Image System (Taichung, Taiwan). Each membrane was re-probed with the antibody against GAPDH that was used as an internal control for equal protein loading. To quantify the protein levels, we used the Image J software (current version1.51n, National Institute of Health, Bethesda, MD, USA).

### 4.9. Preparation of Nuclear Extracts and Measurement of NF-κB Activity

Murine BMDCs (1 × 106 cells/mL) from BALB/c mice, were pre-treated for 2 h with crassolide at various concentrations (1.25–5 μM) and then stimulated for 12 h with 100 ng/mL lipopolysaccharides (LPS). To evaluate NF-κB activity, the cells from BMDCs were used to obtain cytosolic and nuclear extracts using NE-PER (Cat#78833, Thermo Fisher Scientific, Waltham, MA, USA), a nuclear and cytoplasmic extraction reagent. The samples were used to measure NF-κB p65 subunit activation by TransAM NF-κB p65 kit (Cat#40098, Active Motif, Carlsbad, CA, USA) as instructed by each manufacturer’s manual.

### 4.10. Animal Models of Obstetric APS

BALB/c mice were immunized (10 μg/mouse) with β2GPI (Thermo, Tewksbury, MA, USA) along with Freund’s adjuvant (Sigma-Aldrich). Booster injection of 10 μg β2GPI in Freund’s adjuvant was performed for each mouse on day 21. Mice were allowed to mate on day 42. Coital vaginal plugs, indication of successful mating, were counted in the following morning. Fetal losses were calculated on day 56 according to the following formula in percentages: number of absorbed fetuses with respect to the total number of normal and absorbed fetuses [39].

### 4.11. Animal Models of Vascular APS

BALB/c mice were similarly immunized with β2GPI as described above. On day 56, mice were anesthetized with 100 mg/kg intraperitoneal injection of Zoletil^®^. Then sodium fluorescein (60 mg/kg) was infused at the tail vein. Under light microscopy (magnification, ×400), mesenteric venules were irradiated at 520 nm wavelength, and the time taken for vascular occlusion was recorded [40].

### 4.12. Evaluation of Side Effects Relevant to Carssolide Treatment in Mice

To evaluate side effects of carssolide, 5 female BALB/c mice were randomly separated into 4 groups (5/group) and treated with either vehicle as controls or with crassolide (5, 10, or 20 mg/kg). Animals further received similar intraperitoneal doses of crassolide for 34 consecutive days. Afterwards, the animal blood was collected for analysis. Hematological parameters were determined by Neubauer’s chamber, to obtain mean numbers of RBC and WBC. The hepatic function (alanine aminotransferase and alkaline phosphatase) and renal function (urea and creatinine) were also evaluated for crassolide toxicity.

### 4.13. Treatments

Mice were divided into three groups: (a) healthy normal mice (normal control), (b) APS mice receiving intraperitoneal doses of 10% DMSO and 90% glyceryl trioctanoate (vehicle control), and (c) same as group (b) except receiving instead 5 mg/kg of daily crassolide on day 21 till the end of experiment.

### 4.14. Determination of Platelet Count and aPTT

Blood samples were collected from the animal’s orbital sinus on day 56, and anticoagulation-treated with ethylene diamine tetraacetic acid (EDTA). The HEMAVET^®^ reagent kit was used to determine platelet counts on blood smears, using the HEMAVET 950 hematology analyzer (Drew Scientific, Miami Lakes, FL, USA). Collected blood samples underwent further anticoagulation treatment with 3.2% sodium citrate (1 part of sodium citrate to 9 parts of blood), and centrifuged to obtain serum samples. We used a clotting method (Sysmex CA-530, Kobe, Japan) to determine the activated partial thromboplastin time (aPTT).

### 4.15. Analyses of Blood Titer of Anti-β2GPI IgG Antibody

Serum samples were obtained on day 56 and then diluted 1:100 in Tris-buffered saline (pH 8.0) that contained 1% bovine serum albumin (BSA) and 0.5% Tween-20. Anti-β2GPI IgG titers were determined using the ELISA kit (MyBioSource, San Diego, CA, USA) according to the manufacturer’s protocol.

### 4.16. Proliferative Response and Cytokine Production of Murine Spleen Cells after β2GPI Stimulation

On day 56 after primary immunization, mouse spleens were processed to yield single-cell suspensions, by pressing specimen tissues between frosted glass slides and then filtered through a 70 µM nylon mesh. Cells were seeded at a density of 5 × 10^5^ cells/well in 96-well plates, which contained 200 μL of culture medium with 10% fetal calf serum, 50 μg/mL gentamicin, 2 mm glutamine and 50 μM 2-mercaptoethanol. Plates were treated with β2GPI (20 μg/mL) for 96 h. During the final 18 h of incubation, T cell proliferation was measured by the incorporation of [^3^H] thymidine (1 μL Ci/well; NEN-DuPont, Boston, MA, USA). After the total of 96 h of incubation, secreted cytokine levels in culture supernatant were determined using the ELISA kit (eBioscience). The cytokines measured included IFN-γ, IL-17A, and IL-10. For intracellular cytokine productions, mouse spleen cells were treated with Β2GPI for 96 h. The Golgi inhibitor brefeldin A (10 µg/mL) was added to halt further protein production. After washing, splenocytes were stained for surface markers using PerCP-Cyanine5.5-conjugated anti-mouse CD4 (BioLegend, San Diego, CA, USA). Cells were fixed, followed by permeabilization using the Cytofix/Cytoperm Plus Kit (BD Biosciences, San Diego, CA, USA) according to the manufacturer’s instructions. Cells were stained with FITC-conjugated mAbs against murine IFN-γ, IL-17A and Foxp3 (BioLegend). Splenocytes were finally detected by Accuri C5 cytometer and analyzed by C6 Accuri system software (Accuri Cytometers Inc.).

### 4.17. Reverse Transcription-Quantitative Polymerase Chain Reaction (RT-qPCR)

Total RNA was extracted from purified CD11c+ DCs using TRIzol^®^ reagent (Invitrogen; Thermo Fisher Scientific, Inc.). RNA concentrations were determined based on the absorbance of ultraviolet light at 260 nm. Total RNA (2 µg) was reversibly transcribed with moloney murine leukemia virus (MMLV) reverse transcriptase, 5× reaction buffer, dNTPs, RNasin (RNase inhibitor) and oligo (dT) 15 primers (Promega Corporation, city, state abbrev if USA, country), mixed in a total volume of 20 µL. Briefly, incubating at 70 °C, primer annealing was performed for 5 min, followed by the addition of MMLV reverse transcriptase and kept at 37 °C for 60 min. The reverse transcription reaction was terminated by incubation at 72 °C for 10 min. Quantitative PCR was subsequently performed by Fast SYBR™ Green Master Mix (Cat. No. 4385618, Applied Biosystems; Thermo Fisher Scientific, Woolston Warrington, UK) using the ABI 7500 Fast Real-Time system (Applied Biosystems; Thermo Fisher Scientific, Inc.) according to the manufacturer’s protocol. We performed qPCR in the following cycles: initial denaturation at 95 °C for 5 min, followed by 40 cycles of annealing (at 95 °C for 15 s) and extension (at 60 °C for 1 min). The primer sequences used were as follows: IL-12 forward, 5′- GCCAGTACACCTGCCACAAA-3′ and reverse, 5′-TGTGGAGCAGCAGATGTGAGT-3′; IL-23 forward- 5′-GTATCCAGTGTGAAGATGGTTGTGA-3′ and reverse, 5′-CGGA TCCTTTGCAAGCAGAA-3, and hypoxanthine guanine phosphoribosyl transferase 1 (HPRT) forward, 5′-GTTGGATAAGGCCAGACTTTGTTG-3′ and reverse, 5′-GATTCAACTTGCGCCATCTTAGGC-3′ (Tri-I Biotech, Inc., Taipei, Taiwan). Gene expression levels were calculated in relative terms, based on the 2^−∆∆^Cq method. For quantification, the target gene was normalized to the internal standard gene, hypoxanthine guanine phosphoribosyltransferase (HPRT). Data were expressed as multiples or folds relative to the normal group.

### 4.18. Statistical Analyses

Results were expressed as the mean ± SEM. One-way ANOVA test was performed to analyze the data, along with Tukey’s multiple comparison test. We used GraphPad Prism v5.0 software (GraphPad Software; San Diego, CA, USA) for analyses. Statistical significance was set at *p* < 0.05.

## 5. Conclusions

In conclusion, crassolide treatment was beneficial for both vascular and obstetric manifestations of APS while producing no detectable toxicity in mice. Its therapeutic effect was mediated partly through the suppression of DC activation and its downstream Th1 and Th17 responses. Crassolide could be further evaluated as an adjunctive therapy for APS patients.

## Figures and Tables

**Table 1:** Clinical manifestations in mice.

	Normal Mice	Vehicle-Treated APS Mice	Crassolide 5 mg/kg-Treated APS Mice
aPTT (s)	19.3 ± 7.2 ***	91.3 ± 21.4	43.3 ± 23.6 *
Platelet count (1 × 103 c3lls)	673 ± 199 ***	312 ± 183	495 ± 136 *
Anti-β2GPI (O.D. 450 nm)	0.11 ± 0.21 ***	1.96 ± 0.71	0.65 ± 0.42 **
Fetal loss (%)	14 ± 6 *	46 ± 8	23 ± 4 *

The data are presented as the means ± standard deviation of triplicate assays from 6–8 mice/group. * *p* < 0.05, ** *p* < 0.01, *** *p* < 0.001, versus vehicle-treated APS mice, as determined by a one-way ANOVA with Tukey’s multiple comparison test. APS, antiphospholipid syndrome; aPTT, activated partial thromboplastin time; β2GPI, β2-glycoprotein I.

## Data Availability

The data that support the findings of this study are available from the corresponding author upon reasonable request.

## Supplementary Materials

The following are available online. Supplementary Figure S1. Evaluation of side effects of carssolide-treatment in mice. BALB/c mice were treated with crassolide (5, 10, 20 mg/kg per day for consecutive 34 days) and potential side effects were determined. On day 34, blood was collected from control and crassolide-treated mice and hematological parameters (RBC and WBC count), liver function tests (alkaline phosphatase and alanine aminotransferase) and kidney function tests (creatinine and urea) were measured. Data are presented as mean ± SEM of 5 mice. * *p* < 0.05 versus vehicle-treated mice group, tested by one way ANOVA followed by Tukey’s multiple comparison test. Supplementary Figure S2. Expressions of surface markers, CD80 and CD86, of splenic DCs in mice. Flow cytometric analysis of splenic cells in different mouse groups was performed on day 56. The black line represents cells labeled with isotype-matched control antibodies. Supplementary Figure S3. Frequencies of IFN-γ+CD4+ (Th1), IL-17A+ CD4+ (Th17), and Foxp3+CD4+ (Treg) splenic cells in APS mice. Flow cytometric analysis of splenic cells of different mouse groups on day 56.
