# Supplementary material for: Crassolide Suppresses Dendritic Cell Maturation and Attenuates Experimental Antiphospholipid Syndrome
Source: Molecules. 2021 Apr 24;26(9):2492. doi: 10.3390/molecules26092492 (PMC8123116; doi:10.3390/molecules26092492)
Supplement: Supplementary file 1 [file molecules-26-02492-s001.zip › molecules-1137608-SI.pdf]

Supplemental Fig 1

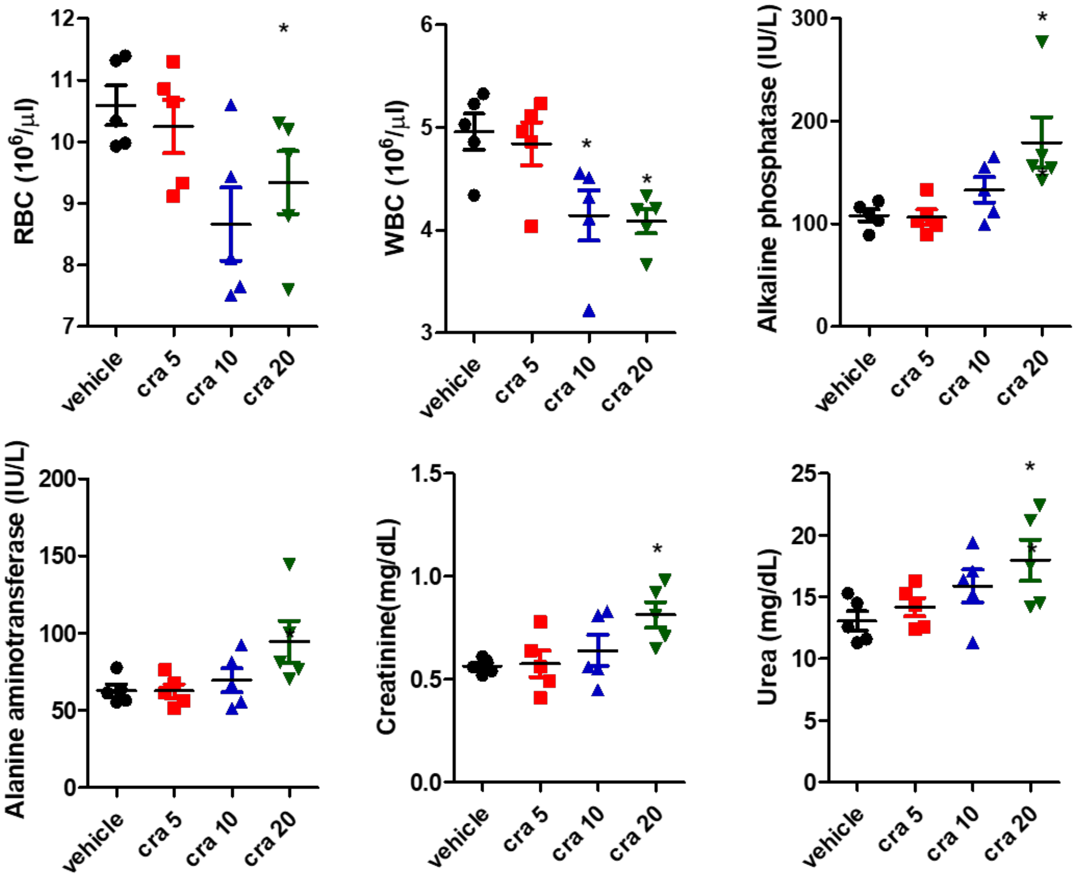

Supplementary Figure S1. Evaluation of side effects of carssolide-treatment in mice. BALB/c mice were treated with crassolide (5, 10, 20 mg/kg per day for consecutive 34 days) and potential side effects were determined. On day 34, blood was collected from control and crassolide-treated mice and hematological parameters (RBC and WBC count), liver function tests (alkaline phosphatase and alanine aminotransferase) and kidney function tests (creatinine and urea) were measured. Data are presented as mean ±SEM of 5 mice. *$p < 0.05$ versus vehicle-treated mice group, tested by one way ANOVA followed by Tukey's multiple comparison test.

Supplemental Fig 2

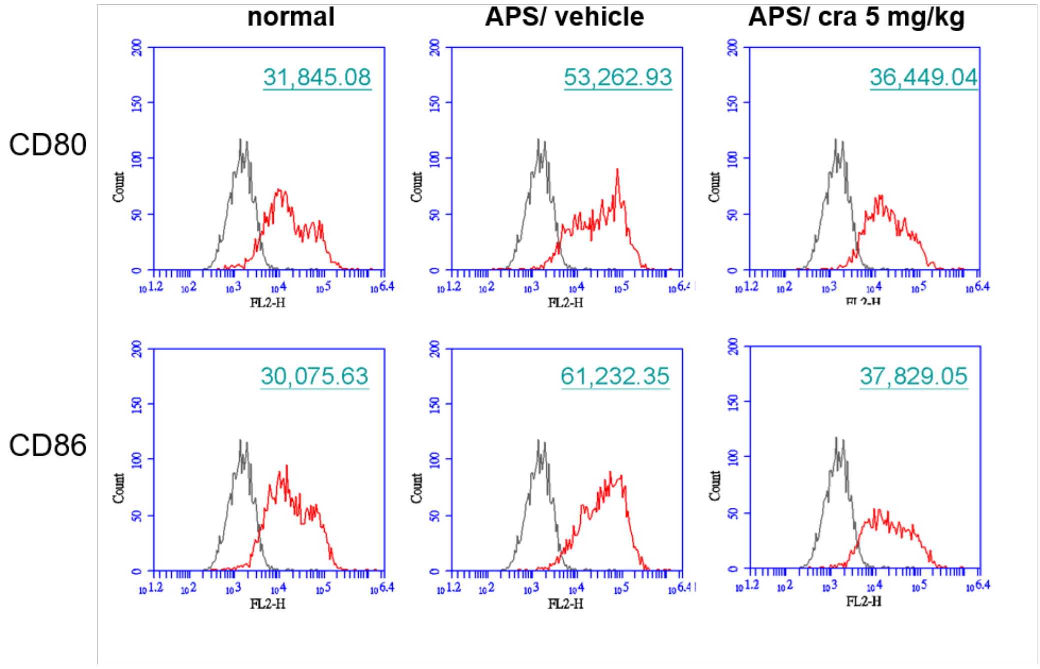

Supplementary Figure S2. Expressions of surface markers, CD80 and CD86, of splenic DCs in mice. Flow cytometric analysis of splenic cells in different mouse groups was performed on day 56. The black line represents cells labeled with isotype-matched control antibodies.

Supplemental Fig 3

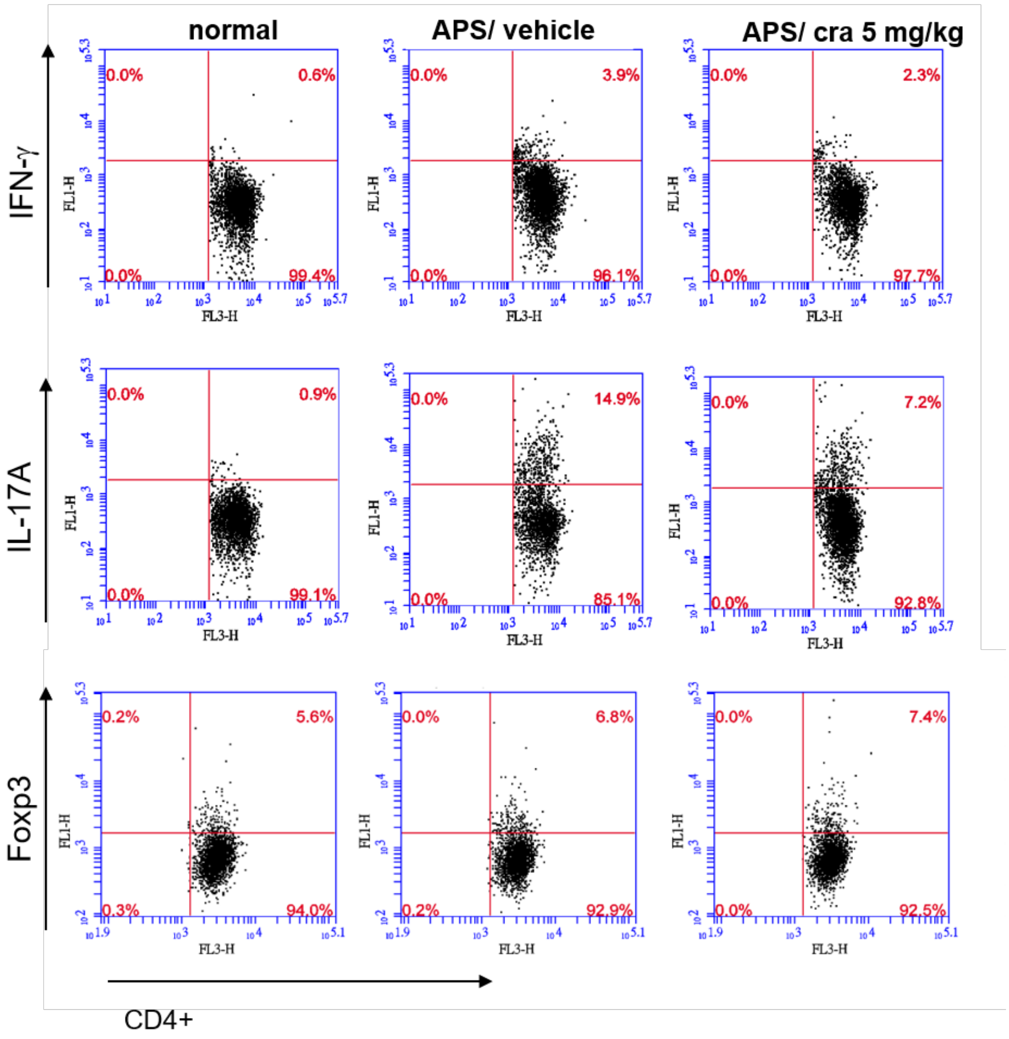

Supplementary Figure S3. Frequencies of IFN-γ+CD4+ (Th1), IL-17A+CD4+ (Th17) and Foxp3+CD4+ (Treg) splenic cells in APS mice. Flow cytometric analysis of splenic cells of different mouse groups on day 56.
